# Addressing Migration Stigma in Latin America Using Mental Health Registry-Based Data

**DOI:** 10.3389/ijph.2026.1608806

**Published:** 2026-04-23

**Authors:** Franco Mascayano, Emily Dunkel, Param Sampat, Katrina M. Rodriguez, Rodrigo Casanueva, Jeanette A. Stingone, Ezra Susser, Lawrence H. Yang

**Affiliations:** 1 Department of Mental Health, Bloomberg School of Public Health, Johns Hopkins University, Baltimore, MD, United States; 2 Institute of Public Health, Universidad Andres Bello, Santiago, Chile; 3 Department of Social and Behavioral Sciences, New York University School of Global Public Health, New York, NY, United States; 4 Department of Epidemiology, Columbia University Mailman School of Public Health, New York, NY, United States; 5 Department of Psychiatry, Columbia University College of Physicians and Surgeons, New York State Psychiatric Institute, New York, NY, United States; 6 School of Nursing, Duke University, Durham, NC, United States; 7 Department of Population Sciences, Duke University, Durham, NC, United States

**Keywords:** migration, stigma, early psychosis, Latin America, population registries

## Abstract

**Background:**

Latin America is experiencing unprecedented migration, with millions, including many political refugees, moving within and across the region. This mass migration carries significant mental health implications due to multi-level stressors, including migration-related stigma.

**Main Text:**

Migration Stigma, in which migrants are labeled as dangerous, criminal, or “other,” drives discrimination and creates structural barriers to mental healthcare, particularly for people with psychosis. This stigma intensifies with intersectionality–factors like ethnicity, gender, or socioeconomic status contribute to greater delays and risk. Although research on migration and psychosis exists–Latin America remains understudied. Population-based registries like Chile’s national registries and Brazil’s 100 Million Cohort offer tools to quantify inequities, identify intervention points, and evaluate policies. Such data can illuminate how stigma and systemic barriers affect care for migrants with psychosis.

**Conclusion:**

Integrating registry-based data with anti-stigma strategies and inclusive health policies is critical to ensuring equitable early psychosis care for Latin American migrants.

Latin America is currently experiencing one of the largest migration flows in its history, involving individuals migrating from, within and into the region. For example, over 7.7 million people have fled Venezuela since 2014, with more than 6.5 million now hosted in Latin American and Caribbean countries [1]. Migration can be both a cause and consequence of severe stressors – including poverty, overcrowding, disrupted social networks and loss of fundamental resources like housing and employment – that adversely affect mental health [2]. Critically, migration status itself often (but not always) becomes a stigmatizing label that elicits a host of stigmatizing consequences. Stigma is known to severely undermine mental health outcomes by creating barriers to care, social support, and recovery [3]. In the context of early psychosis – a time-limited window where prompt intervention can improve long-term outcomes – such barriers can be especially detrimental.

Researchers have coined the term “migration stigma,” which highlights how migrants are broadly labeled with undesirable characteristics, such as being dangerous or engaged in criminal activities. Yang et al. [4] describe a process whereby being labeled “migrant” triggers stereotyping, social “othering,” and discrimination, resulting in loss of social status in the receiving society. Structural stigma can arise when laws or policies treat migrants as second-class (e.g., restricting their access to health services). Migrants may also internalize negative societal messages and avoid seeking health or social services due to shame or fear [4, 5]. Although this concept is primarily grounded with examples from the U.S. and Europe, similar processes occur in Latin America, where powerful nationalist or xenophobic rhetoric increasingly frames migrants as burdens or outsiders.

There is a robust global literature showing that migrants (and their children) have higher incidence of psychotic disorders than host populations. For instance, large meta-analyses indicate that migrants are on average about twice as likely to develop schizophrenia or related psychoses [6]. This elevated risk is thought to be driven largely by social determinants, including social adversity, discrimination, and the stress of migration [7]. Furthermore, a Swedish national cohort found that migrants living in neighborhoods with fewer co-ethnics had higher risk of non-affective psychosis [8]. Specifically, each 5% decrease in “own-region” migrant density was associated with a 5% higher hazard of psychosis in first- and second-generation migrants.

How does this relate to Latin America? Latin America has its own migration dynamics–including rural-to-urban migration, internal displacement, and flows from within the region (e.g., Haitians to higher income Latin American settings). Research on migration and psychosis in Latin America is sparse, but what does exist suggests similar patterns of vulnerability. For example, in Chile – which has become a significant destination country –migrants show worse mental health outcomes than the native-born population [9]. Qualitative research in the region also notes high rates of trauma, stress, and discrimination among migrants, all of which can precipitate or worsen psychotic symptoms [10]. Moreover, the UN Refugee Agency has found that increasing competition for jobs and limited access to public services have led to cases of discrimination and xenophobia towards Venezuelan migrants in South America [1].

One promising avenue is to harness registry-based data for mental health research [11]. High-income countries have long used administrative registries to study psychiatric disorders in entire populations, including migrants. These data allow researchers to link an individual’s migration background to their mental health diagnoses and service use over time. Latin America is now beginning to build similar capacities. For example, the Chilean Registries, which contain records for approximately 95% of the entire Chilean population including migrants, offer reliable, population-level documentation on a range of social determinants of health related to migrant status and clinical indicators. These registries include the only national registry of individuals with first episode psychosis (FEP) worldwide, providing the opportunity to examine the association of migrant status and other social determinants with illness onset, progression, and mental health services use for individuals with psychosis.

Chile’s National FEP Registry, which includes information on migrants and their countries of origin, captures at which level of care individuals with FEP are first identified (e.g., primary care vs. specialized services)––and enables investigations concerning the confluence of migration status and other social determinants. This information can provide valuable insight into the populations and sites where future assessments of stigma at individual and structural-levels and other barriers are most needed to decrease treatment delays. In unpublished work from our group, we found migrants with FEP were more likely than Chilean nationals to be identified in hospital and emergency settings. That migrants with FEP are more likely to access care during acute crises suggest substantial barriers and prolonged duration of untreated psychosis, highlighting the need for culturally targeted interventions, grounded in the migration stigma framework, to facilitate earlier engagement with care.

Brazil also offers opportunities for research into migration stigma, such as the 100 Million Brazilian Cohort, created by the government, which links the national social-protection registry (Cadastro Único) with mortality, hospital, infectious-disease and birth databases for >130 million low-income residents, allowing researchers to flag both international migrants and internal migrants and to follow mental-health outcomes and service use across the life course [12]. Elsewhere, Colombia’s integrated health information platform SISPRO unifies hospital, outpatient and insurance claims and records patient nationality in the Individual Service Registry, enabling analyses of service use and psychiatric admissions among migrants versus locals [13]. Finally, PAHO’s regional R4V Health & Psychosocial Support dashboard collates standardized clinic-level data on refugee and migrant consultations (diagnosis, service type, nationality) from 17 Latin-American countries, illustrating that even where full population registries are just emerging, pragmatic surveillance systems already capture migrant mental health encounters [14].

Thus, a registry-based approach could quantify what is currently anecdotal: the mental health disparities faced by migrants. The high-dimensional nature of the data would allow assessment of potential effect modifiers over time (e.g., social deprivation), and testing of hypotheses derived from stigma theory. Importantly, registries can assess impacts of reducing structural stigma: for instance, by evaluating the impact of a policy change (such as granting legal residency) on mental health service usage among migrants. In Europe, longitudinal registers revealed that when migrant-dense communities provided social support, psychosis risk decreased. Latin American registries could be used similarly, to evaluate whether anti-discrimination laws or regional-level programs have measurable effects [8].

Understanding migration stigma in early psychosis has clear policy relevance. PAHO emphasizes the integration of migrant health needs into national policies, and PAHO offers toolboxes for Mental Health and Psychosocial Support (MHPSS) in migration crises [14]. However, mental health is often overlooked in migration policy. For instance, while some countries have implemented standard programs to document migrants’ health and grant access to services [1], there may be few specific provisions for mental healthcare. Mexico’s General Health Law (2012) and Mental Health Law (2017) enshrine the right to mental healthcare for everyone, but implementation for migrants remains inconsistent. Brazil’s successful “Psychosocial Law” and network of community mental health centers have expanded access, but they too must be adapted to culturally diverse migrants.

At the health system level, it is essential to raise providers’ competence in addressing social determinants of health and stigma related to migration. Interventions could include training clinicians in migrant trauma caused by dislocation, anti-bias workshops, and community outreach in migrant neighborhoods. Registry findings could inform such efforts: if data show, for example, that migrants enter care later in the course of illness or more often involuntarily (as seen in some European studies of migrants with psychosis) [15], targeted policies could be devised (e.g., outreach by early psychosis teams in immigrant communities).

Finally, legal and social policies play a decisive role in shaping migration stigma in early psychosis, and population-based registries offer a powerful means to evaluate their real-world impact. By linking policy reforms—such as changes in legal status, entitlement to health services, or anti-discrimination protections—to patterns of service access, crisis presentations, and duration of untreated psychosis, registries can reveal whether governments are effectively reducing structural stigma. Regional examples such as Colombia’s 2018 temporary protection statute for Venezuelans and Chile’s regularization programs extending legal residency to recent migrants illustrate how registries can assess whether expanded rights translate into earlier engagement with care. Investing in robust registries and integrating them into policy planning allows countries to monitor unintended consequences, test equity-focused interventions, and identify communities facing persistent barriers.
